# Piperaquine concentration and malaria treatment outcomes in Ugandan children treated for severe malaria with intravenous Artesunate or quinine plus Dihydroartemisinin-Piperaquine

**DOI:** 10.1186/s12879-019-4647-2

**Published:** 2019-12-03

**Authors:** Pauline Byakika-Kibwika, Ronald Ssenyonga, Mohammed Lamorde, Daniel Blessborn, Joel Tarning

**Affiliations:** 1 Department of Medicine, Makerere University College of Health Sciences, Kampala, Uganda; 2 Infectious Diseases Institute, Kampala, Uganda; 3 Clinical Trials Unit, Makerere University College of Health Sciences, Kampala, Uganda; 4 Mahidol-Oxford Tropical Medicine Research Unit, Mahidol University, Bangkok, Thailand; 5 Centre for Tropical Medicine, Nuffield Department of Medicine, University of Oxford, Oxford, UK

**Keywords:** Piperaquine, Pharmacokinetics, Malaria, Children

## Abstract

**Background:**

Treatment for severe malaria must be prompt with effective parenteral antimalarial drugs for at least 24 h to achieve fast parasite clearance, and when the patient can tolerate oral therapy, treatment should be completed with effective artemisinin based combination therapy (ACT) for complete parasite clearance and to prevent recrudescence. We evaluated piperaquine concentration and malaria treatment outcomes among Ugandan children treated for severe malaria with intravenous artesunate (AS) or quinine (QN) plus dihydroartemisinin-piperaquine (DP), in Tororo District Hospital in Eastern Uganda.

**Methods:**

Capillary blood piperaquine concentration data were obtained from a randomized clinical trial whose objective was to evaluate parasite clearance, 42-day parasitological treatment outcomes and safety, following treatment of severe malaria with intravenous AS or QN, plus artemether-lumefantrine or DP among children in Tororo District Hospital, in Eastern Uganda.

**Results:**

Piperaquine concentration data from 150 participants who received DP were analyzed. Participants with unadjusted treatment failure had lower median day 7 capillary piperaquine concentration than those with treatment success (34.7 (IQR) (17.9–49.1) vs 66.7 (IQR) (41.8–81.9), *p* < 0.001), and lower than the recommended day 7 cut off level of 57 ng/mL. There was no difference in median capillary piperaquine concentrations among participants with re-infection and recrudescence (35.3 (IQR) (17.9–55.2) vs 34.8 (IQR) (18.1–45.1), *p* = 0.847). The risk of treatment failure was two times higher among children with low (< 57 ng/mL) day 7 capillary piperaquine concentration (relative risk: 2.1 CI 1.4–3.1), *p* < 0.001) compared to children with high day 7 capillary piperaquine concentrations (> 57 ng/mL).

**Conclusion:**

Considering the low day 7 concentrations of piperaquine reported in the patients studied here, we suggest to adopt the recently recommended higher dose of DP in young children or a prolonged 5-day dosing in children living in malaria endemic areas who have suffered an initial episode of severe malaria in order to achieve adequate drug exposures for effective post-treatment prophylactic effects.

**Trial registration:**

The study was registered with the Pan African Clinical Trial Registry (PACTR201110000321348). Registered 7th October 2011.

## Background

Severe malaria is a life threatening emergency, responsible for 435,000 deaths annually, worldwide, with the greatest burden in sub-Saharan Africa [1]. Treatment for severe malaria must be prompt with effective parenteral antimalarial drugs for at least 24 h to achieve fast parasite clearance, and when the patient can tolerate oral therapy, treatment should be completed with effective artemisinin based combination therapy (ACT) for complete parasite clearance and to prevent recrudescence. Uganda adopted the policy to use intravenous artesunate (AS) as first line treatment for severe malaria in 2013, with intravenous quinine (QN) or intramuscular artemether as alternatives. The oral ACTs; artemether-lumefantrine (AL) and dihydroartemisinin-piperaquine (DP) are recommended for complete parasite clearance [2–4]. Although previous studies demonstrated excellent effectiveness of both AL and DP for treatment of uncomplicated malaria, DP provides the additional advantage of once daily dosing and longer post treatment prophylactic effect of up to 35 days compared to AL, which is dosed twice daily and with a shorter post treatment prophylactic period of 28 days [3, 5, 6]. We evaluated capillary piperaquine concentration and malaria treatment outcomes among Ugandan children treated for severe malaria with intravenous AS or QN plus DP, in Tororo District Hospital in Eastern Uganda.

## Methods

### Study design, site and population

Study methodology has been previously described and published as a randomized single blind clinical trial conducted in Tororo District Hospital in Eastern Uganda [7], an area with perennial malaria transmission and an annual entomological inoculation rate estimated to be 310 infective bites per person per year [8]. The main study enrolled consecutive patients aged 6 months and above, with signed informed consent provided by the parent or guardian and severe malaria defined as presence or history of fever plus a positive blood film for *P.falciparum* malaria, with at least one of the laboratory or clinical features of severe malaria. Patients were excluded if they had obvious concomitant febrile illness, history of allergy to any of the study drugs, if they could not comply with study procedures and visits, or if they had received an antimalarial drug within 24 h before presenting to hospital.

### Treatment

Intravenous AS (Guilin Pharmaceutical Factory, Guangxi, China) was administered as a slow bolus into an indwelling cannula as 2.4 mg/kg at start of treatment, repeated at 12 and 24 h and every 24 h till the switch to oral therapy. Intravenous QNN dihydrochloride (Rotex, Trittau, Germany) was administered over 4 h as 10 mg/kg body weight in 5% dextrose (10 ml/kg) and repeated 8 hourly till the switch to oral therapy.

Parenteral antimalarial therapy was administered for at least 24 h, followed by a full course of the oral ACT when participants could tolerate oral therapy. Oral AL (Coartem, Novartis, 20 mg artemether/120 mg lumefantrine tablets) was administered according to body weight as; one (5–14 kg), two (15–24 kg), three (25–34 kg) and four (> 35 kg) tablets 12 hourly, with a cup of milk or food, for 3 days. Oral DP (Eurartesim, Sigma-Tau, dihydroartemisinin (DHA) 40 mg + piperaquine (PQP) 320 mg tablets) was administered targeting a total dose of 6.4 and 51.2 mg/kg of dihydroartemisinin and piperaquine, respectively, given in three equally divided doses to the nearest quarter tablet. We used a pill cutter to ensure that the tablet fractions were as close to the nearest quarter tablet as possible.

All participants received oral paracetamol in a dose of 15 mg/kg at 8 hourly intervals. Adjunctive and supportive treatment for complications of malaria such as convulsions and hypoglycemia was given in accordance with the Uganda Ministry of Health guidelines. The study nurse provided information to caretakers about adherence to drugs, follow-up visits and potential drug side effects. Caretakers were instructed to observe the participants for 30 min after drug administration and if vomiting occurred they were to administer another dose, for up to two extra doses, following which they were to bring back the participant to the study clinic for evaluation and treatment.

### Follow-up

We performed serial blood smears at 0, 1, 2, 4, 6, 8, 10, 12, 16, 20, 24 h post start of intravenous therapy and every 6 h until 6 h post parasite clearance. Participants were initiated on oral ACT and discharged from hospital when they could tolerate oral ACT and the blood smear was negative for malaria parasites, and were followed up for 42 days to ascertain parasitological outcomes and monitor adverse events on days 1, 2, 3, 7, 14, 21, 28, 35, 42, and any unscheduled day if the participant felt unwell. On each of these days we took medical history and performed physical examination, a finger prick was done to collect blood on slides for malaria diagnosis and on filter paper for genotyping and drug concentration measurements. Participants with positive malaria films were reassessed for severity and treated accordingly, those with severe malaria were re-admitted and treated with intravenous AS plus AL, and those with uncomplicated malaria were evaluated for treatment failure and treated according to national guidelines. Participants were discontinued from study follow up if they could not take study medication, missed a scheduled follow-up visit and could not be located at home, or if they received non study drugs.

### Laboratory procedures

Thin smears were performed to determine the type of malaria parasite species and thick smears for parasite density. Thick blood smears were stained using 3% Giemsa for 30 min and read by two independent experienced laboratory technologists, blinded to participants’ treatment assignment. Any discrepant results were reviewed by a tie breaker.

Parasite density was calculated by counting the number of asexual parasites (ring stages) per 200 white blood cells (WBCs) or per 500 if the count was less than 10 parasites per 200 WBCs, assuming a WBC count of 8000/uL of blood. A smear was considered negative if no parasites were seen after review of 100 high-power fields. Complete blood count and hemoglobin estimation were performed using the Coulter counter (Beckman coulter, Life Science, United States of America).

Molecular genotyping of paired samples was conducted to distinguish re-infection from recrudescence, at the Makerere University-University of California San Francisco Molecular Biology laboratory in Mulago, Kampala. We used Whatman 3MM filter paper from Sigma. Parasite DNA was extracted from filter paper blood samples collected on the day of enrollment and the day of parasitological treatment failure using Chelex 100 Resin extraction (Bio-Rad Laboratories, Hercules, CA) as previously described [9]. The surface antigen loci MSP1, MSP2 and GLURP were amplified using previously described primers [10]; 2 μL of template DNA was amplified using nested polymerase chain reaction (PCR), with second round primers specific to allelic families: K1, MAD20, and RO33 for msp1, msp2 and the repeat region of glurp [11]. PCR products were stained with ethidium bromide separated by electrophoresis on a 2.5% agarose gel (UltraPure Agarose; Invitrogen, Carlsbad, CA). GelCompar II software (Applied Maths, Sint-Martens-Latem, Belgium) was used to select alleles and estimate the size of PCR products using a standardized approach [12]. Recrudescence was defined as the presence of all matched alleles on day 0 and the day of failure at every locus and reinfection defined as at least one locus showing unmatched alleles.

### Classification of outcomes

The primary study outcome was parasitological treatment failure unadjusted by genotyping classified as parasitemia detected by thick blood smear. This primary outcome was selected because it best represents the treatment outcome measure used in routine clinical care. The secondary outcomes were parasitological treatment failure adjusted by genotyping classified as reinfection or recrudescence. Adverse events were defined as any medical occurrence post study drug administration. They were graded as mild, moderate, severe and life threatening and their relationship to the study drug was classified as unrelated, possibly, probably or definitely related to study drug.

### Piperaquine concentration measurement

On each follow up day ie days 1, 2, 3, 4, 7, 14, 21, 28, 35, 42 post the commencement of ACT administration, capillary blood samples were collected by finger prick and stored dry on Whatman 3MM filter paper. The fingers were disinfected and pricked with a lancet, following which the first blood drop of blood was discarded and the next three drops collected. Each drop filled a pre marked circle on filter paper. The filter papers were allowed to dry at room temperature and packed in sealed ziplock bags.

The blood samples were transported at room temperature, to the Department of Clinical Pharmacology at the Mahidol-Oxford Tropical Medicine unit, Faculty of Tropical Medicine, Mahidol University, Bangkok, Thailand. Piperaquine concentrations were measured using an LC-MS/MS based assay and validated according to U.S. FDA guidelines (unpublished data). Briefly, 3 discs of 3.2 mm diameter were punched out from each dried blood spot sample and 375 μL stable isotope internal standard in phosphate buffer 50 mmol/L pH 2.0 was added followed by 150 μL perchloric acid (0.3 mol) and 75 μL acetonitrile and then mixed for 60 min. Approximately 500 μL was transferred to a 96 well plate solid phase extraction column and extracted with a MPC-SD Empore 96-wellplate standard well 1 ml (3 M Empore, 3 M Centre, St. Paul, MN, USA). The extracted sample was then evaporated until dry and reconstituted in 250 μL acetonitrile-ammonium bicarbonate 2.5 mmol/L pH 10 (85–15 v/v). The LC-MS/MS assay settings were the same as a previously published method [13]. The lower limit of quantification was 3 ng/ml and triplicate quality control samples at low, medium and high concentration was included in each batch of samples to ensure accuracy and precision of the assay. The total coefficient of variation for all quality control samples were within the acceptance criteria of the U.S. FDA guidelines for sample analysis.

### Ethical considerations

The study was approved by Makerere University School of Medicine Research and Ethics Committee (REC REF 2011–175), Uganda National Drug Authority (369/ESR/NDA/DID-12/2011), Uganda National Council for Science and Technology (HS 1031) and registered with the Pan African Clinical Trial Registry (PACTR201110000321348). All study procedures were conducted according to Good Clinical Practice standards. Patients and parents or guardians of participants provided written informed consent prior to enrollment. Study related information was provided in the participants’ local languages.

### Statistical analysis

Data were entered and verified using MS ACCESS and analyzed using STATA version 13.1 (STATA Corporation, College Station, TX, USA). Descriptive statistics were used to compare demographic and clinical characteristics among the four study arms. Continuous variables were compared using Wilcoxon test for non-normally distributed data. Categorical variables were compared using Chi-square test. Parasite density was normalized using logarithmic transformation. Intention-to-Treat analysis was used for comparison of treatment outcomes, which included all enrolled participants. Unadjusted treatment failure was classified as a positive blood smear on any of the follow-up days. Adjusted treatment failure was classified as either re-infection or recrudescence based on genotyping. The risk of treatment failure at 28, 35 and 42 days of follow up (unadjusted and adjusted by genotyping) were estimated using the Kaplan-Meier survival method and compared using the Log Rank test. Time at risk was calculated from day one of ACT allocation to date of treatment failure among participants who failed, last day of follow-up for those who did not complete follow-up, or day 42 for the patients who completed 42 days of follow-up. In the analysis for adjusted parasitological outcomes, only recrudescence was considered as true parasitological treatment failure. Safety data from all participants were analyzed.

Piperaquine concentration data were compared using the Wilcoxon test. We compared day 7, 14, 35 and 42 piperaquine concentration among children with and without malaria treatment failure. Day 7 piperaquine concentration were also stratified above/below a previously reported cut off level of 57 ng/mL, associated with an increased risk of therapeutic failure [9], and evaluated with respect to risk of malaria treatment failure.

## Results

We enrolled and followed up 300 participants between January 2012 and March 2013, of whom, 150 received DP. Baseline characteristics were similar across the four treatment arms (Table 1). Adverse events occurred commonly, although most were of mild to moderate severity and consistent with malaria symptoms. The most common were headache, nausea and vomiting. All severe adverse events were classified as unrelated to study drugs and all were treated and resolved completely.

**Table 1:** Baseline characteristics of study participants

Characteristic	AS+DP *N* = 79	AS+AL *N* = 71	QN + DP N = 71	QN + AL *N* = 79
Female (%)	36 (45.6)	36 (50.7)	24 (33.8)	37 (46.8)
Age in months*	17 (11–26)	16 (10–26)	17 (13–26)	18 (13–26)
Weight (kgs)*	9.5 (8–11.5)	9 (8.1–11)	9.8 (8.8–11)	9.2 (8.4–11)
Temperature (degrees Centigrade)*	38.8 (37.7–39.5)	39.1 (37.7–39.5)	39.1 (37.3–39.5)	38.6 (37.5–39.6)
Parasite density per uL, log10 copies*	4.82 (4.29–5.10)	4.79 (4.38–5.02)	4.72 (4.19–5.00)	4.73 (4.17–5.03)
Complications at admission, n (%)				
Hemoglobin (mg/dL)*	9.1 (7.9–10.5)	9.2 (8.4–10.6)	9.3 (8.4–10.4)	9.4 (8.0–10.3)
Total white blood cell count (*10^3/^uL)*	9.6 (6.9–13.2)	9.2 (7.5–12.3)	9.4 (7.7–12.1)	10.0 (7.0–14.3)
Random blood sugar (mmol/L)*	7.3 (6.3–8.3)	6.8 (6.4–8.3)	6.8 (5.7–8.3)	7.4 (6.25–8.25)
History of repeated convulsions n (%)	6 (7.6%)	3 (4.2%)	8 (11.3%)	1 (1.3%)
History of inability to feed	26 (33.0%)	24 (34.0%)	22 (31.0%)	29 (36.7%)
Prostration (extreme weakness)	22 (27.9%)	15 (21.13%)	21 (29.58%)	21 (26.58%)
Hemoglobinuria	0	0	2 (2.8%)	0
Jaundice	2 (2.5%)	0	3 (4.2%)	3 (3.8%)
Severe anemia	0	0	1 (3%)	2 (2.5%)
Respiratory distress	2 (2.5%)	6 (8.5%)	3 (4.2%)	5 (6.3%)
Impaired consciousness	0	0	1 (1.4%)	0
Abnormal bleeding	2 (2.5%)	1 (1.4%)	0	1 (1.3%)
Hypoglycemia	0	0	0	1 (1.3%)

### Piperaquine concentration

Capillary piperaquine concentration data from the 150 participants who received DP were analyzed. Of these, 17 had treatment outcome assignment before day 7, and 133 were followed up past day 7. Median (IQR) day 7 capillary piperaquine concentration was 42.1 (23.2–67.2) ng/mL, lower than the recommended cut off level of 57 ng/mL [9], and 90 (67.7%) had capillary piperaquine concentration less than 57 ng/mL.

The observed median (IQR) day 7 capillary piperaquine concentrations were significantly lower in patients with recrudescence (34.8 ng/mL (IQR) (18.1–45.1) and re-infection (35.3 ng/mL (IQR) (17.9–55.2) compared to patients with successful malaria treatment outcome (66.7 ng/mL (IQR) (41.8–81.9), both *p* < 0.001).

**Fig. 1:**
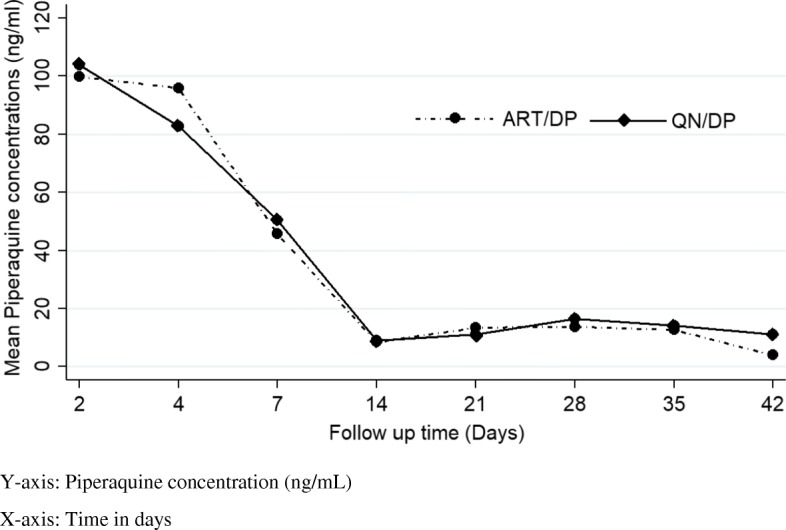
Mean (SD) capillary piperaquine concentration by study day and arm

Furthermore, the risk of treatment failure (recrudescence and re-infection) was two times higher (relative risk: 2.1, 95% CI: 1.4–3.1, *p* < 0.001) among children with low (< 57 ng/mL) day 7 capillary piperaquine concentrations compared to children with high day 7 capillary piperaquine concentrations (> 57 ng/mL). Figure 1 shows the observed capillary piperaquine concentrations on each study day, stratified by study arm and Table 2 shows capillary piperaquine concentrations stratified by treatment outcome and follow up day.

**Table 2:** Capillary piperaquine concentration, stratified by treatment outcome and day

Day	Median (IQR) capillary piperaquine concentration (ng/mL)					
	Unadjusted treatment outcomes			Adjusted treatment outcomes		
	Treatment Success (*n* = 52)	Treatment Failure (*n* = 98)	*p*	Re-infection (*n* = 63)	Recrudescence (*n* = 35)	*p*
2	126 (61.4–215.0)	58.4 (30.3–105.0)	0.002	61.5 (34.3–105)	61.5 (35.8–94.3)	0.939
4	110.5 (77.5–155.5)	66.7 (32.4–103.0)	< 0.001	72.8 (38.8–106)	59.4 (24.3–98.9)	0.300
7	66.7 (41.8–81.9)	34.7 (17.9–49.1)	< 0.001	35.3 (17.9–55.2)	34.8 (18.1–45.1)	0.847
28	15.3 (11.3–23.6)	10.8 (7.6–14.3)	0.001	11.4 (7.3–16.7)	14.3 (8.2–18.1)	0.549
35	12.1 (8.2–5.6)	10.4 (5.0–15.4)	0.352	7.9 (4.9–11.1)	15.4 (13.2–22.2)	0.095

## Discussion

The objective of this study was to evaluate piperaquine concentration and malaria treatment outcomes among Ugandan children treated for severe malaria with intravenous AS or QN plus DP, in Tororo District Hospital in Eastern Uganda. The oral ACT, DP was effective at clearing parasites during the 42-day follow-up period, with good safety outcomes. There were low rates of recrudescence, majority of the study patients classified as treatment failures suffered re-infection with malaria parasites during the follow-up period [7]. Our findings are consistent with previous data from similar high malaria transmission settings which demonstrated high rates of re-infection with malaria parasites after initial antimalarial treatment [2, 10, 11]. The high malaria re-infection rate is particularly important in patients with severe malaria since re-infection is likely to cause further co-morbidity and resulting in negative health and social-economic impact.

Piperaquine is the long acting partner drug in the DP combination and is responsible for clearing residual parasites to prevent recrudescence while also preventing re-infection (post treatment prophylaxis). Previous studies have reported day 7 piperaquine capillary concentration of 57 ng/mL as a therapeutic target, with lower concentrations associated with an increased risk of recrudescence in patients treated for uncomplicated malaria [14]. In 2015 Sambol et al. reported that less than 30% of Ugandan children receiving weight based dosing of piperaquine for uncomplicated malaria achieved 57 ng/mL on day 7 [15]. Our findings are in agreement with this as only 32.3% of our patients achieved more than the target of 57 ng/mL.

We found that children with piperaquine concentration below this target had an approximately 2-fold higher risk of malaria treatment failure. Despite this, we demonstrated low levels of recrudescence among our study participants with no difference in the risk of recrudescence across study arms [7].

Previous studies have demonstrated high risk for readmission or death within 6 months’ post discharge among children hospitalized with severe malaria in malaria endemic areas. Therefore, researchers have recommended administration of malaria chemoprevention with DP at discharge in order to protect from novel infections in the period following the severe malaria episode [16–18]. Treatment with DP offers superior post-treatment prophylaxis compared to AL, due to the longer terminal elimination half-life of piperaquine leading to therapeutic concentrations for an extended period of time compared to lumefantrine [11].

## Conclusion

Considering the low day 7 concentrations of piperaquine reported in the patients studied here, we suggest to adopt the recently recommended higher dose of DP in young children [19] or a prolonged 5-day dosing in children living in malaria endemic areas who have suffered an initial episode of severe malaria in order to achieve adequate drug exposures for effective post-treatment prophylactic effects.

## Data Availability

The datasets used and/or analyzed during the current study are available from the corresponding author on reasonable request.

## Abbreviations

ACT: Artemisinin based combination therapy
AL: Artemether-lumefantrine
AS: Artesunate
DHA: Dihydroartemisinin
DP: Dihydroartemisinin-piperaquine
IQR: Inter Quartile Range
PCR: Polymerase chain reaction
PQP: Piperaquine
QN: Quinine
WHO: World Health Organization
